# Public perception of facial vascularized composite allotransplants-insights from a cross-sectional survey of healthy individuals in the USA

**DOI:** 10.1016/j.jpra.2025.11.015

**Published:** 2025-11-16

**Authors:** Leonard Knoedler, Thomas Schaschinger, Raffaele Aguglia, Curtis L. Cetrulo, Max Heiland, Carsten Rendenbach, Rakan Saadoun, Felix J. Klimitz, Martin Kauke-Navarro, Bohdan Pomahac, Jakob Fenske, Alexandre G. Lellouch

**Affiliations:** a Department of Oral and Maxillofacial Surgery, Charité – Universitätsmedizin Berlin, Corporate Member of Freie Universität Berlin and Humboldt-Universität zu Berlin, Augustenburger Platz 1, 13353 Berlin, Germany; b Division of Plastic and Reconstructive Surgery, Cedars-Sinai Medical Center, Los Angeles, CA, USA; c Vascularized Composite Allotransplantation Laboratory, Center for Transplantation Sciences, Massachusetts General Hospital, Harvard Medical School, Boston, MA, USA; d Department of Otolaryngology, University of Texas Health Science Center at Houston, Houston, TX, USA; e Department of Surgery, Division of Plastic and Reconstructive Surgery, Yale New Haven Hospital, Yale School of Medicine, New Haven, CT, USA; f Université Paris Cité, Inserm, The Paris Cardiovascular Research Center, Team Endotheliopathy and Hemostasis Disorders, Paris, France; g Hematology Department, AP-HP, Hôpital Européen Georges Pompidou, Paris, France

**Keywords:** Face transplant, Facial vascularized composite allotransplantation, Allograft, Vascularized composite allotransplantation, VCA, Public survey

## Abstract

**Background:**

Facial vascularized composite allotransplantation (fVCA) is an advanced reconstructive procedure for extensive facial defects. While clinical outcomes have improved, public perceptions remain underexplored. Gaining insights into societal attitudes is crucial for patient education, informed decision-making, and guiding future research.

**Methods:**

A cross-sectional survey was conducted among 100 volunteers from the United States via the Prolific platform. The study population reflected the demographic distribution of the general population. The survey assessed awareness, willingness to undergo fVCA, decision-making factors, and ethical concerns. Responses were collected through Likert scales, multiple-choice questions, and aesthetic evaluations of post-transplant outcomes.

**Results:**

Fifty-eight percent of participants had never heard of fVCA, with social media being the most common source of information (50 %) for those who had. Eighty-seven percent were potentially open to undergoing fVCA in case of an accident, although concerns about graft rejection risks (65 %) and lifelong immunosuppression (65 %) influenced decision-making. Functionality was prioritized over aesthetics by 51 % of respondents, yet 72 % preferred donor-recipient matching based on age, gender, and ethnicity. Psychological adaptation was a major concern for 51 %, with 82 % emphasizing the importance of pre- and postoperative psychological counseling. Ethical concerns included donor consent (61 %) and fairness in allocation (69 %). Respondents rated photographs of postoperative aesthetic outcomes with a mean score of 7.9 out of 10.

**Conclusion:**

While public perception of fVCA is generally positive, concerns regarding rejection, immunosuppression, and psychological adaptation remain significant. Increased education, ethical transparency, and advancements in immunosuppressive strategies are needed to enhance acceptance and feasibility. Future research should explore novel immunosuppressants and psychological programs to improve long-term concerns.

## Graphical abstract

**Figure:**
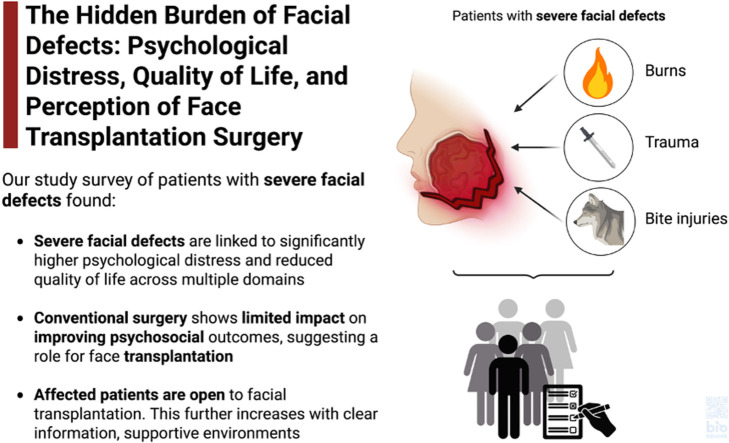

## Introduction

Facial vascularized composite allotransplantation (fVCA) represents one of the most complex and groundbreaking recent surgical achievements in reconstructive surgery, with >50 procedures performed globally to date.^1^^,^^2^ These surgeries have transformed the lives of patients suffering from severe facial deformities due to trauma, burns, or congenital conditions, substantially improving their functional outcomes, psychological health, and social reintegration.^1^^,^^3^^,^^4^

Facial transplantation involves the transplantation of facial tissue from a deceased donor to a recipient, restoring both appearance and critical functions such as speaking, eating, and facial expressions and sensation.^5^, ^6^, ^7^, ^8^, ^9^ The surgical procedure can be resource-intensive and challenging, requiring meticulous planning,^10^ precise anatomical matching, and extensive postoperative care to mitigate complications like rejection, infection, and functional impairments.^11^^,^^12^

Despite clinical advancements and improved surgical outcomes, public perception and awareness of facial transplantation remain poorly understood. Previous research has primarily focused on clinical and technical outcomes, leaving a critical gap in understanding societal attitudes and potential stigmas associated with this transformative procedure. However, understanding public perception is vital because it supports the reintegration of future transplant recipients into the community, psychological well-being, and guides future research.^13^^,^^14^

The current study addresses this gap by exploring public opinions and concerns about facial transplantation, focusing on factors such as aesthetic outcomes, functional recovery, psychological adjustment, and ethical considerations. By surveying a diverse sample of respondents, we attempt to understand broader societal attitudes toward facial transplantation, including preferences for donor-recipient matching, awareness and acceptance of immunosuppressive risks, and concerns about psychological, functional, and aesthetic complications. The insights derived from our findings have important implications for future public education initiatives, clinical guidelines, and policymaking to improve patient outcomes and societal acceptance of facial transplantation.

## Methods

### Study design and survey structure

The institutional review board at Cedars-Sinai Medical Center (Los Angeles, CA, USA) reviewed and approved this cross-sectional survey (IRB Exemption). In total, 64 survey questions (single and multiple choice, Likert scales, numerical rating scale (NRS)) were designed covering various aspects of fVCA. A short introduction paragraph on the topic was presented. On participant level, demographics and medical background were determined. For gender determination, the two-step method described by Lagos and Compton was applied.^15^ On procedural level, participants were asked about various attitudes towards fVCA procedures concerning functional and aesthetical preferences, willingness to undergo surgery and personal financial engagement, immunosuppressive therapies and psychological aspects. Lastly, participants were shown publicly available photographs of five patients before and after fVCA surgery and asked to rate the aesthetic result on a numerical rating scale (NRS) from 1 to 10, with 1 indicating “completely inadequate result” and 10 indicating “perfect result.” While originally developed for pain assessment, NRS-like scales have also been used in prior studies to capture subjective aesthetic impressions in a straightforward and sensitive manner.^16^^,^^17^ In this study, we adapted the item wording to ensure clarity and accessibility for a general audience, aiming to facilitate intuitive and consistent responses from participants with diverse background. Supplementary Digital Content 1 provides a comprehensive overview of all survey questions and answer options.

All questions were integrated in a Google Forms (Google LLC, Mountain View, CA, USA) spreadsheet. Subsequently, the survey was registered on the Prolific (Prolific Academic Ltd., London, UK) commercial survey platform. A standard sample of 100 voluntary participants, who had to be at least 18 years old and US residents, were recruited to complete the survey anonymously. Eligible participants were required to have fluent English proficiency to ensure accurate comprehension of survey questions and responses. Participants also needed access to a computer or mobile device with a stable internet connection to complete the questionnaire in full. Only those who provided informed consent before beginning the survey were included in the final dataset. Participants were excluded if they submitted incomplete surveys, failed attention-check questions, or reported taking the survey multiple times. Automated, duplicate, or low-quality responses identified by Prolific’s internal verification system were also excluded to maintain data integrity and reliability. Upon successful completion, participants were compensated equally by the Prolific platform. Based on the CIOMS International Ethical Guidelines for Biomedical Research, participants received $13.41 per hour for completing the questionnaire 18. Due to the internal structure of the Prolific survey platform, personal data of all participants were completely deidentified and not available for researchers at any time point. The survey was conducted in March 2025. The complete questionnaire is attached in Supplement Material 1. The manuscript was checked against the Strengthening the Reporting of Observational Studies in Epidemiology (STROBE) checklist (Supplemental Appendix).

### Data analysis

Survey results were stored and managed using spreadsheet software (Microsoft Excel Version 16.56, Microsoft Corp., Redmond, WA, USA). All questions were answered by each participant, leaving no missing data. Descriptive statistics for all questions were obtained using the Python programming (Python Software Foundation Wilmington, DE, USA) language.

## Results

### Study cohort

In total, 100 participants participated in the survey. The response rate was 100 % (*n* = 100 %). Most participants were between 35–44 years (*n* = 30; 30 %), with female sex assigned at birth (*n* = 67; 67 %), identifying as female (*n* = 64; 64 %) and of White ethnicity (*n* = 65; 65 %). The majority of participants resided in the Southern US region (*n* = 37; 37 %). Sixteen percent (*n* = 16) of participants had a background in healthcare, with 4 % (*n* = 4) being healthcare professionals with expertise in plastic and reconstructive surgery, dermatology, aesthetic medicine and/or biomedical engineering. Most participants held a bachelor’s degree (*n* = 47; 47 %). Table 1 details participant demographics and characteristics.

**Table 1:** Participant’s demographics and characteristics.

Demographics	Participants (*n* = 100)	
Gender		
Female	64	(64 %)
Male	33	(33 %)
Non-binary	3	(3 %)
Ethnicity		
White	65	(65 %)
Black or African-American	19	(19 %)
Asian	7	(7 %)
Hispanic/Latino	6	(6 %)
Other	3	(3 %)
Age (in years)		
18–24	7	(7 %)
25–34	23	(23 %)
35–44	30	(30 %)
45–54	23	(23 %)
55–64	12	(12 %)
65+	5	(5 %)
Educational background		
High school or less	29	(29 %)
Bachelor’s degree	47	(47 %)
Graduate or professional degree	20	(20 %)
Medical or healthcare professional	4	(4 %)
US Region		
South	37	(37 %)
Midwest	25	(25 %)
West	20	(20 %)
Northeast	18	(18 %)

### Awareness and knowledge of fVCA

Among all participants, 58 % (*n* = 58) had never heard of fVCA. Social media (*n* = 27; 27 %) and television (*n* = 13; 13 %) were the primary sources for participants who had heard about the procedure. Concerning immunosuppression and rejection, 65 % (*n* = 65) were slightly aware or unaware of immunosuppressive side effects and rejection risks.

### Willingness to undergo fVCA procedures

The majority of respondents expressed openness to undergoing fVCA procedures, particularly when recommended by experts, with 87 % (*n* = 87) considering the option. While many (*n* = 38; 38 %) were hesitant due to the risks of rejection, 65 % (*n* = 65) indicated they might still proceed despite these concerns.

The prospect of lifelong immunosuppressive therapy was acceptable to 65 % (*n* = 65) of participants, provided that the side effects remained mild. Of these 65 %, 26.2 % (*n* = 17) were aware or extremely aware of associated immunosuppressive risks, while 69.2 % (*n* = 45) were slightly or not at all aware. Among those deciding that the risks would outweigh the benefits (16 %; *n* = 16), 25 % (4 %) were aware, while 75 % (*n* = 12) were slightly or not at all aware of associated risks. Finally, among those opting for immunosuppressive therapy regardless of side effects (19 %; *n* = 19), 47.7 % (*n* = 9) were aware, while 42.1 % (*n* = 8) were unaware of associated risks. More than half (*n* = 51; 51 %) showed caution in their decision, weighing the potential complications such as rejection.

Additionally, 43 % (*n* = 43) were willing to relocate to specialized centers to receive fVCA. Optimizing quality of life played a crucial role in decision-making, with 57 % (*n* = 57) preferring either full or partial fVCA based on the best possible outcome. Efforts to minimize multiple surgeries were important to 52 % (*n* = 52) of respondents, while 37 % (*n* = 37) expressed willingness to self-pay up to $10,000 if insurance did not cover the procedure. Patience in finding the most suitable donor was also a key factor, with 48 % (*n* = 48) willing to wait as long as necessary.

### Key decision-making factors

As for aesthetic concerns, 37 % (*n* = 37) were most concerned about an unnatural appearance, with 72 % (*n* = 72) preferring a donor match in age, gender, and ethnicity and 48 % (*n* = 48) deeming symmetry and alignment most important, while acknowledging some asymmetry as acceptable if facial functions were restored (51 %, *n* = 51).

Functional priorities emerged as equally important (*n* = 66; 66 %), with 51 % (*n* = 51) valuing functional restoration (eating, speaking, breathing) over appearance, 56 % (*n* = 56) possibly willing to accept aesthetic compromises for better function and 47 % (*n* = 47) deeming facial sensation and appearance equally important. Fourty-eight percent (*n* = 48) were especially concerned about lagophthalmos. Fifty-seven percent (*n* = 57) would perceive fVCAs as successful if it allowed for the expression of subtle emotions.

As for psychological and social aspects, 51 % (*n* = 51) anticipated an adjustment period of psychological challenges, 31 % (*n* = 31) were extremely concerned about identity challenges, 44 % (*n* = 44) were somewhat concerned about public reactions but were ready to adjust, and 82 % (*n* = 82) deeming pre- and postoperative psychological counseling extremely important.

Most participants believed ethical considerations (e.g., donor consent, fairness in allocation) should play a major role (*n* = 61; 61 %), with 69 % (*n* = 69) valuing shared decision making between patient and surgeon on the procedures. Lifelong immunosuppression (*n* = 35; 35 %) and identity adjustments (*n* = 32; 32 %) were considered the biggest ethical challenges. In the future, 49 % (*n* = 49) would prefer a 3D-printed or bioengineered alternative, if available.

**Figure 1:**
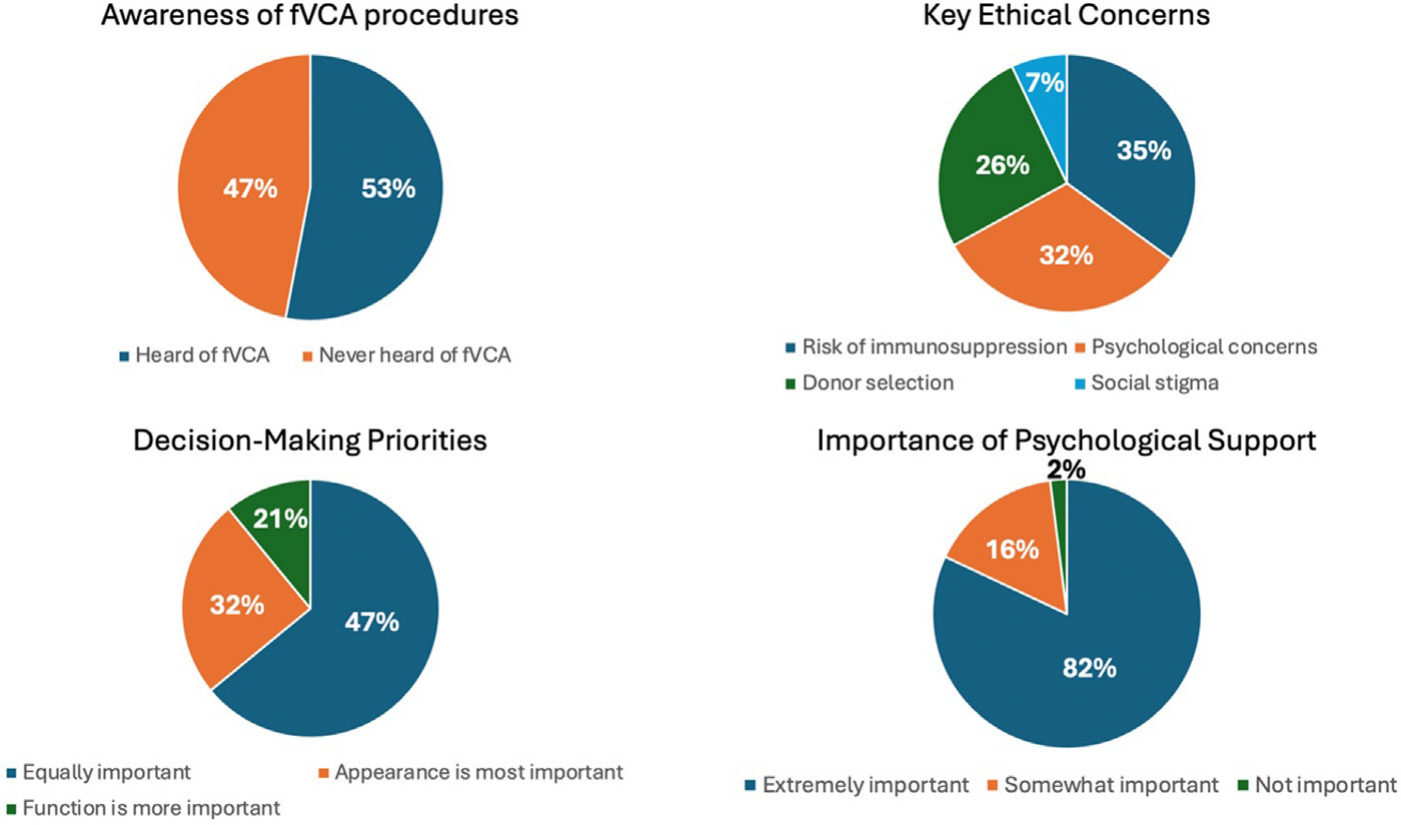
Key results on procedural awareness, ethical concerns, decision-making priorities and importance of psychological support in the public perception.

Overall, participants rated the outcomes of fVCA photographs with a mean of 7.9 ± 1.6 on the NRS. Figure 1 provides an overview of the key findings. Supplementary Digital Content 2 provides an overview of all answers including frequencies for all questions.

## Discussion

Face transplant surgeries are one of the most promising procedures to address extensive composite facial defects with the surgical need projected to increase in the future.^18^^,^^19^ However, data on potential recipients’ and the general public’s perception of fVCA remains scarce and limited to sub-populations and potential donors.^20^, ^21^, ^22^ This cross-sectional survey reveals insights into broader public attitudes, concerns, and key decision-making factors for fVCA procedures, offering insights for VCA patients and providers.^23^

The survey’s results revealed a balance between optimism and apprehension in willingness to undergo fVCA and associated procedures. Eighty-seven percent of respondents were principally open to fVCAs, but a significant proportion also remained hesitant due to rejection risks, revealing that people are both intrigued by the potential of the procedure and wary of its medical uncertainties. As for accompanying systemic therapy, 65 % were willing to commit to lifelong immunosuppressive therapy while keeping their consent conditional on mild side effects. However, it must be noted that the majority of these participants (69.2 %) were only slightly aware or unaware of associated risks of immunosuppression, thereby potentially slightly skewing the consequences that can be drawn from this observation. Similarly, significant portions of participants who opted for immunosuppression in regardless of side-effects (42.1 %) or no immunosuppression at all (62.5 %) were not fully aware of potential risks, leading to comparable interpretations. Although subject to extensive research, rejection episodes remain a significant challenge, with 85 % of patients experiencing at least one acute rejection episode and almost 50 % multiple episodes within the first year.^24^, ^25^, ^26^ Further, four out of nine patients developed signs of clinical and histopathologic chronic rejection over a median follow-up period of 120 months in a recent cohort study, highlighting the importance of ongoing research into tailored immunosuppressive strategies to break down deadlocks and make the procedure more accessible.^8^^,^^27^

Since fVCA procedures are currently not available nationwide, patients are required to accept high mobility requirements. The willingness of 43 % to relocate for the procedure speaks to the perceived value of fVCAs for those in need but also raises questions about healthcare accessibility and equity in specialized surgical centers. Although not directly surveyed in this study, international discrepancies in fVCA availability remain a challenge as well. While the United States has multiple specialized centers,^28^ technologically advanced countries like Germany and Switzerland lack established fVCA programs. This limited structural integration and availability of fVCA underscores the need for healthcare policymakers to improve access and meet medical and public demands. Indeed, when interviewed about VCAs, responses from professionals in organ procurement organizations highlight a disparity in training related to VCAs. The majority of these professionals have never received any formal training on VCAs, and three-quarters report a need for such education.^29^

Besides healthcare policies and treatment modalities, psychological and ethical factors present significant challenges to the routine implementation of fVCAs.^30^ While 51 % expected an adjustment period, 31 % were deeply concerned about losing their self-identity, highlighting the interplay between psychological resilience and identity challenges. When asked, 84 % of ethicists consider face transplantation to be ethically acceptable, although unresolved concerns remain, particularly regarding sex-mismatched and skin tone–mismatched fVCAs.^31^ Consequently, there seems to be a need for robust periprocedural psychological counseling,^32^^,^^33^ which 82 % deemed crucial. This aligns with previously reported outcomes of relevant psychological challenges in fVCA patients.^34^^,^^35^ More specifically, a previous study exploring public attitudes toward VCA showed that while public support for VCA donation is generally favorable, many individuals express discomfort with donating visible body parts, reflecting underlying identity and bodily integrity concerns.^36^ To address this, tailored psychological programs are mandatory in the previously mentioned specialized centers.^37^ On the other hand, public reaction is a secondary concern, with 44 % expressing apprehension but feeling prepared to adapt.^30^ While concerns about social stigma seem to be a factor, self-perception appears to be more critical in decision-making. Moreover, donor consent, fairness in allocation, and life-long immunosuppression^38^ were frequently cited concerns regarding ethical considerations. In summary, there is a future need for transparent policies that align with public expectations and ethical best practices, as well as novel immunosuppressive methods.

Regarding fVCA outcomes, there is a complex balance between aesthetic and functional considerations. While 51 % of respondents prioritized function over appearance, with 56 % willing to accept aesthetic compromises for better functionality, 72 % preferred a donor match in age, gender, and ethnicity, and 48 % deemed symmetry most important. Especially the ability to express subtle emotions was deemed most important for a natural appearance. This near-equal emphasis on aesthetics and function underscores the desire for both aspects to be optimized, revealing the ongoing tension between medical feasibility and public expectations. Moreover, given that current aesthetic outcomes of fVCA are already rated positively (7.9 of 10 points on the NRS),^4^ further advancements in functional outcomes may enhance public satisfaction, potentially establishing fVCA as the gold standard for reconstructing severe facial defects.

## Limitations

While this study helps bridge knowledge gaps and enhances engagement in the field of fVCA surgery, its findings should be interpreted with caution due to several limitations. First, the sample size of 100 participants is relatively small, which may have led to an underrepresentation of attitudes from other segments of society. Second, the study’s findings are specific to the US healthcare system and societal context, limiting their generalizability to other countries. However, it is worth noting that the majority of fVCA procedures to date have been performed in the US.^39^ Third, the self-reported nature of the survey introduces the potential for social desirability bias and personal biases. Factors such as limited real-world exposure to fVCA patients and media influence may have shaped participants’ responses. Results only provide cross-sectional opinions and it cannot be ruled out that these may be altered once participants are confronted with such procedures or defects themselves. Fourth, the cross-sectional study design restricts the ability to assess how attitudes may evolve, for example, following further education about fVCA. Lastly, participants recruited through survey platforms may differ systematically from the general public – for instance, they may have higher research experience or literacy – introducing a potential selection bias.

## Conclusion

This study provides insights into public perceptions of fVCA, highlighting a complex interplay between optimism, caution, and ethical considerations. The findings underscore the necessity of improving public awareness, refining immunosuppressive protocols, and expanding psychological support frameworks to enhance patient acceptance and outcomes. Ethical considerations, including donor matching preferences and procedural fairness, remain critical to fostering trust and societal acceptance. As surgical and biomedical advancements continue to refine fVCA, a multidisciplinary approach integrating public education, healthcare policy adaptations, and technological innovations will be essential in shaping the future of this transformative procedure.

## Funding

None.

## Conflicts of interest

None declared.

## Appendix Supplementary materials

Image, application 1

Image, application 2
