# Supplementary material for: Public perception of facial vascularized composite allotransplants-insights from a cross-sectional survey of healthy individuals in the USA
Source: JPRAS Open. 2025 Nov 16;48:123–31. doi: 10.1016/j.jpra.2025.11.015 (PMC12719695; doi:10.1016/j.jpra.2025.11.015)
Supplement: Supplementary file 1 [file mmc1.docx]

**Supplementary Digital Content 1.** Complete survey with questions and answer choices.

Introduction paragraph: Thank you for participating in this survey on public perceptions of facial transplants (Facial Vascularized Composite Allotransplants, fVCAs). The goal of this survey is to understand what characteristics people prioritize or care about most when it comes to facial transplants. Your responses will remain anonymous. The survey should take approximately 10 to 15 minutes to complete. By proceeding, you consent to participate in this research survey.

1. What is your Prolific ID? (Note: mandatory question on anonymous and deidentified IDs on Prolific)
2. Age (select one)
   1. 18-24
   2. 25-34
   3. 35-44
   4. 45-54
   5. 55-64
   6. 65+
3. Sex assigned at birth (select one)
   1. Male
   2. Female
4. Gender (select one)
   1. Male
   2. Female
   3. Non-binary/other
5. Race/Ethnicity (select one)
   1. White
   2. Black or African-American
   3. Asian
   4. Hispanic or Latino
   5. Other
6. Geographic Location (select one)
   1. **Northeast** (Maine, New Hampshire, Vermont, Massachusetts, Rhode Island, Connecticut, New York, Pennsylvania, New Jersey)
   2. **Midwest** (East North Central: Ohio, Indiana, Illinois, Michigan, Wisconsin or West North Central: Minnesota, Iowa, Missouri, North Dakota, South Dakota, Nebraska, Kansas)
   3. **South** (South Atlantic: Delaware, Maryland, Virginia, West Virginia, North Carolina, South Carolina, Georgia, Florida or East South Central: Kentucky, Tennessee, Alabama, Mississippi or West South Central: Arkansas, Louisiana, Oklahoma, Texas)
   4. **West** (Mountain: Montana, Idaho, Wyoming, Nevada, Utah, Colorado, Arizona, New Mexico or Pacific: Washington, Oregon, California, Alaska, Hawaii)
7. Have you ever heard of facial Vascularized Composite Allotransplants (FVCAs)?
   1. No
   2. Yes, on social media
   3. Yes, on television
   4. Yes, from peer-groups
   5. Yes, other
8. Have you read or seen information about facial transplant procedures?
   1. No
   2. Yes, on social media
   3. Yes, on television
   4. Yes, from peer-groups
   5. Yes, other
9. Have you or someone close to you experienced severe facial trauma or undergone reconstructive surgery?
   1. Yes
   2. No
10. Do you have a background in healthcare?
    1. Yes
    2. No
11. **Educational Background**
    1. High school or less
    2. Bachelor’s degree
    3. Graduate or professional degree
    4. Medical or healthcare professional
12. **Do you have expertise in any of the following fields?** (Check all that apply)
    1. Plastic and Reconstructive Surgery
    2. Dermatology
    3. Aesthetic Medicine
    4. Biomedical Engineering
    5. Psychology / Psychiatry
    6. General Public (no medical background)
13. If you required a face transplant to restore function and appearance, what is the maximum amount you would be willing to pay out-of-pocket for the procedure, assuming costs are not covered by insurance?
    1. $0 – I would only proceed if fully covered by insurance
    2. $1 – $10,000
    3. $10,001 – $50,000
    4. $50,001 – $100,000
    5. $>100,000
14. If you were a candidate for a face transplant, how long would you be willing to wait for a suitable donor and the procedure?
    1. <1 month
    2. 1-3 months
    3. 3-6 months
    4. 6 months-1year
    5. 1-2 years
    6. As long as it takes to find the best match
15. If you were to receive a facial transplant, which aesthetic complications would bother you the most?
    1. Facial asymmetry
    2. Visible or excessive scarring
    3. Mismatch in skin tone or texture
    4. Loss of facial expression or muscle movement
    5. Swelling or prolonged facial distortion
    6. An unnatural or artificial appearance
    7. Differences in hair growth (e.g., color, texture, or patchy areas)
    8. Changes in lip or eyelid positioning
    9. None of these would bother me
16. Would you prefer a face transplant from a donor who closely matches your age, gender, and ethnicity?
    1. Yes, all three factors should match
    2. Age and gender should match, but ethnicity is not important
    3. Only gender should match
    4. Matching is not important to me
17. Which functional complications after a face transplant would concern you the most? (Select up to three)
    1. Difficulty speaking clearly
    2. Difficulty eating or drinking normally
    3. Loss of sensation in the face
    4. Loss of ability to smile or express emotions
    5. Difficulty breathing
    6. Chronic pain or discomfort
    7. None of these would bother me
18. Are you aware of the risks associated with facial transplants, such as rejection or the need for lifelong immunosuppression?
    1. Extremely aware
    2. Aware
    3. Neutral
    4. Slightly aware
    5. Not at all aware
19. Would you be willing to take lifelong immunosuppressive medications (therapy that suppresses the immune system) with potential side effects to maintain a facial transplant?
    1. Yes, regardless of side effects
    2. Yes, but only if side effects are mild
    3. No, the risks outweigh the benefits
20. If you were to receive a face transplant, how important would donor-recipient resemblance be to you?
    1. Extremely important
    2. Somewhat important
    3. Neutral
    4. Not important at all
21. How would you feel about the possibility of facial rejection, which could require additional surgeries or removal of the transplant?
    1. I would accept the risk and still undergo the procedure
    2. I would be hesitant but might still consider the procedure
    3. I would not undergo the procedure if rejection was a significant possibility
22. If given the option, would you prefer a partial or full facial transplant?
    1. Full face transplant for optimal function and appearance
    2. Partial face transplant to minimize surgical risk
    3. Whichever option maximizes my quality of life
23. How important is maintaining your pre-transplant identity when receiving a new face?
    1. Extremely important—I want to look as much like my old self as possible
    2. Somewhat important—I’d like some resemblance but accept changes
    3. Neutral—I just want a functional and natural-looking face
    4. Not important—I would be fine with a completely different appearance
24. Would you feel comfortable sharing your face transplant journey publicly (e.g., media interviews, social media, medical studies)?
    1. Yes, I would be open to sharing my story
    2. Only in medical or scientific settings
    3. No, I would prefer to keep it private
25. If you underwent a face transplant, how concerned would you be about societal acceptance and public reactions?
    1. Extremely concerned—I worry about stigma and negative reactions
    2. Somewhat concerned—I anticipate challenges but would adjust
    3. Neutral—I don’t think it would be a major issue
    4. Not concerned at all—I would embrace my new appearance
26. If you needed a face transplant, how much influence would your family or loved ones have on your decision?
    1. A great deal—they would need to support the decision
    2. Somewhat—I would consider their opinions but make my own choice
    3. Very little—I would decide independently
    4. None—my decision would be entirely my own
27. If you required a face transplant, how much of a priority would restoring facial sensation be compared to appearance?
    1. Sensation is more important than appearance
    2. Appearance is more important than sensation
    3. Both are equally important
28. How does symmetry of facial movement impact the perception of success in a facial transplant?
    1. Critical—both static and dynamic symmetry must be achieved
    2. Some asymmetry is acceptable if function is restored
    3. Movement symmetry is secondary to overall facial volume and contour
29. How concerned would you be about developing psychological or identity challenges after a face transplant?
    1. Extremely concerned—it would be difficult to adjust
    2. Somewhat concerned—I anticipate an adjustment period
    3. Neutral—I would take it as it comes
    4. Not concerned at all—I would fully embrace the change
30. Would you be willing to undergo multiple surgeries over several years to optimize the outcome of your face transplant?
    1. Yes, as many as necessary to achieve the best result
    2. Yes, but I would prefer to limit the number of procedures
    3. No, I would want to minimize additional surgeries
31. If a face transplant was your only option to restore function and appearance, but it was considered experimental with unknown long-term outcomes, would you proceed?
    1. Yes, I would take the risk
    2. Maybe, depending on the available research and expert recommendations
    3. No, I would not want to undergo an experimental procedure
32. What aspect of a face transplant would be the most important in your decision-making?
    1. Restoring normal function (e.g., eating, speaking, breathing)
    2. Achieving a natural appearance
    3. Minimizing risk of rejection and complications
    4. Avoiding lifelong immunosuppressive medications
    5. The psychological and social impact
33. Would you consider a face transplant if it meant you would never fully regain facial movement?
    1. Yes, appearance alone would be enough
    2. Yes, but I would hope for some movement recovery
    3. No, I would not undergo the procedure without functional improvement
34. If a 3D-printed or bioengineered alternative to a face transplant became available, would you prefer it over a donor transplant?
    1. Yes, if it reduces risks like rejection and immunosuppression
    2. No, I would prefer a donor face for a more natural result
    3. I would consider whichever option had the best long-term outcomes
35. How much does the potential for long-term complications (e.g., chronic rejection, infections) influence your willingness to undergo a face transplant?
    1. Significantly—I would be hesitant due to the risks
    2. Somewhat—I would proceed with caution
    3. Not much—I would accept the risks for the benefits
    4. Not at all—The benefits outweigh the potential risks
36. Would you be willing to relocate to receive a face transplant at a specialized center if it was not available in your current location?
    1. Yes, I would relocate anywhere necessary
    2. Yes, but only within a reasonable distance
    3. No, I would only undergo the procedure if it was available locally
37. How much of a role should ethical considerations (e.g., donor consent, fairness in organ allocation) play in deciding whether someone receives a face transplant?
    1. A major role—ethical concerns should heavily influence decisions
    2. Some role—ethics are important but not the main deciding factor
    3. A minor role—the medical need should take priority
    4. No role—I would not consider ethical concerns in my decision
38. How important is it for face transplant recipients to receive psychological counseling before and after surgery?
    1. Extremely important—it should be required for all recipients
    2. Somewhat important—it should be offered but not required
    3. Not important—I don’t think it’s necessary
39. Would you be willing to have your transplanted face appear noticeably different from your original appearance if it meant better function?
    1. Yes, function is more important than appearance
    2. Maybe, but I would prefer some resemblance to my original face
    3. No, I would not want a face that looks too different
40. If face transplants became more common and widely accepted, how do you think public perception of recipients would change?
    1. They would become more accepted and normalized
    2. They would still face stigma and curiosity
    3. There would be no significant change in public perception
41. How much influence should surgeons have in deciding whether someone qualifies for a face transplant?
    1. Surgeons should have the final say based on medical criteria
    2. There should be shared decision-making between surgeons and patients
    3. Patients should have the final say, even if they don’t meet all medical criteria
42. If you underwent a face transplant, how would you prefer to see yourself for the first time?
    1. Alone, in private
    2. With close family or friends for support
    3. With medical professionals to guide the experience
43. Which of the following factors do you think would matter the most for a successful facial transplant outcome?
    1. Skin tone matching
    2. Facial volume (fullness and shape)
    3. Symmetry and alignment
    4. Skin texture
    5. Scar visibility
44. How important is it to you that the skin tone of the transplanted face closely matches the recipient’s original skin tone? (Scale of 1-5, where 1 = Not important, 5 = Extremely important)
    1. 1
    2. 2
    3. 3
    4. 4
    5. 5
45. How important is achieving natural facial volume (e.g., avoiding puffiness or hollowness) in facial transplants? (Scale of 1-5, where 1 = Not important, 5 = Extremely important)
    1. 1
    2. 2
    3. 3
    4. 4
    5. 5
46. Do you think slight asymmetry (e.g., one side of the face is slightly different from the other) affects the perceived success of a facial transplant?
    1. Strongly agree
    2. Agree
    3. Neutral
    4. Oppose
    5. Strongly oppose
47. Do you think it’s more important for a facial transplant to focus on:
    1. Functionality (ability to smile, speak, eat)
    2. Appearance (aesthetics)
    3. Both equally
48. Would you consider a face transplant as a valid option for individuals with severe facial deformities or injuries?
    1. Yes, it provides a better quality of life
    2. Maybe, depending on the results
    3. No, I think it’s too risky
49. What are your thoughts on the use of facial transplants to help restore the appearance of individuals with severe trauma, burns, or congenital deformities?
    1. Strongly support
    2. Support
    3. Neutral
    4. Oppose
    5. Strongly oppose
50. How do you feel about the use of facial transplants for purely cosmetic reasons rather than medical necessity?
    1. Strongly support
    2. Support
    3. Neutral
    4. Oppose
    5. Strongly oppose
51. If you needed a facial transplant, how important would it be to you that the donor face closely matches your original appearance? (Scale of 1-5, where 1 = Not important, 5 = Extremely important)
    1. 1
    2. 2
    3. 3
    4. 4
    5. 5
52. How comfortable would you feel interacting with someone who has undergone a facial transplant? (Scale of 1-5, where 1 = Not comfortable and 5 = Very comfortable)
    1. 1
    2. 2
    3. 3
    4. 4
    5. 5
53. In your opinion, how well do facial transplant patients reintegrate into society after their surgery?
    1. Very well
    2. Somewhat well
    3. Not well
    4. Unsure
54. How important do you think public education is in helping the general population understand facial transplants?
    1. Very important
    2. Important
    3. Neutral
    4. Not important
55. Which aspect of a facial transplant do you think would be the biggest challenge for the recipient?
    1. Social acceptance
    2. Psychological adjustment to a new face
    3. Functional aspects (e.g., speech, eating, blinking)
    4. Risk of rejection and medical complications
56. Which of the following post-transplant facial movement deficits would be most concerning?
    1. Inability to fully close eyes (lagophthalmos)
    2. Limited smile and perioral movement
    3. Lack of forehead or eyebrow mobility
    4. Difficulty with speech articulation
57. Would you perceive a transplanted face as more ‘normal’ if it maintains the ability to express subtle emotions?
    1. Yes, microexpressions and dynamic movement are essential
    2. No, static symmetry is more important than movement
58. What level of skin texture difference between transplanted and native facial skin is acceptable?
    1. No noticeable difference
    2. Mild roughness or scarring is acceptable
    3. Moderate differences are acceptable if pigmentation is uniform
    4. Texture mismatches are not a major concern
59. What do you think is the greatest ethical challenge in facial transplantation?
    1. Identity and psychological adjustment for the recipient
    2. Donor selection and consent process
    3. Risk of immunosuppression and long-term complications
    4. Social stigma and public perception
60. How successful do you rate the result of the operation? (1=completely inadequate, 10=perfect) (Link: <https://de.pinterest.com/pin/510384570247778055/>, Source: CBS News)


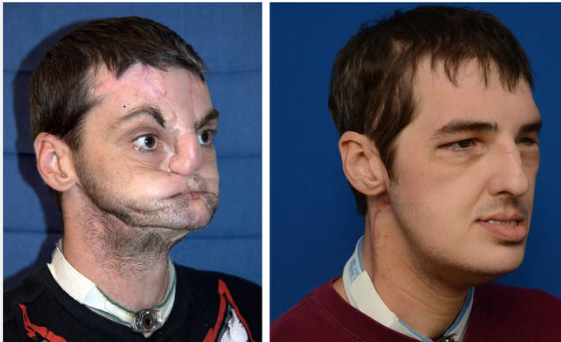


- 1. 1
  2. 2
  3. 3
  4. 4
  5. 5
  6. 6
  7. 7
  8. 8
  9. 9
  10. 10

1. How successful do you rate the result of the operation? (1=completely inadequate, 10=perfect) (Link: <https://hospitalnews.com/first-canadian-face-transplant-a-success/>, Source: Hospital News)


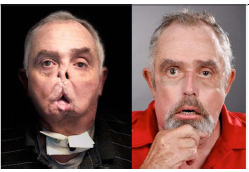


- 1. 1
  2. 2
  3. 3
  4. 4
  5. 5
  6. 6
  7. 7
  8. 8
  9. 9
  10. 10

1. How successful do you rate the result of the operation? (1=completely inadequate, 10=perfect) (Link: <https://www.3d-grenzenlos.de/magazin/kurznachrichten/3d-druck-macht-gesichtstransplantation-moeglich-27233393/>, Source: 3D GRENZENLOS)


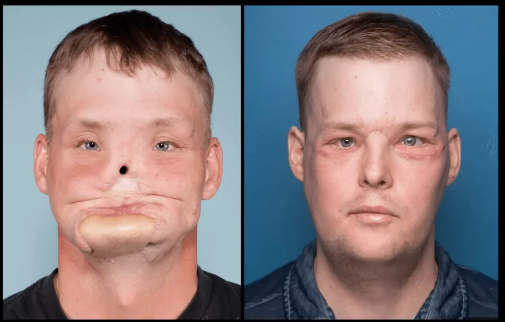


- 1. 1
  2. 2
  3. 3
  4. 4
  5. 5
  6. 6
  7. 7
  8. 8
  9. 9
  10. 10

1. How successful do you rate the result of the operation? (1=completely inadequate, 10=perfect) (Link: <https://www.plasticsurgery.org/for-medical-professionals/publications/psn-extra/news/planning-the-key-to-highly-complicated-and-successful-face-transplant>, Source: American Society of Plastic Surgeons)


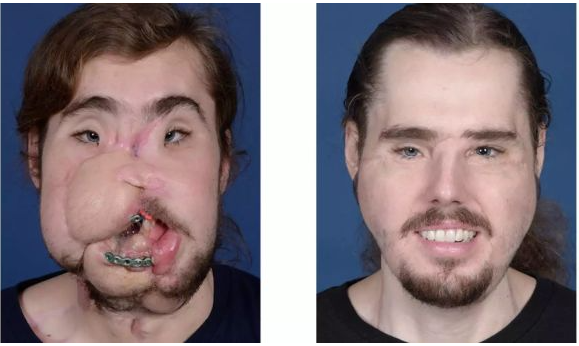


- 1. 1
  2. 2
  3. 3
  4. 4
  5. 5
  6. 6
  7. 7
  8. 8
  9. 9
  10. 10

1. How successful do you rate the result of the operation? (1=completely inadequate, 10=perfect) (Link: <https://www.faz.net/aktuell/gesellschaft/gesundheit/gesichtstransplantation-ich-habe-meine-nase-wieder-1795266.html>, Source: Frankfurter Allgemeine Zeitung)


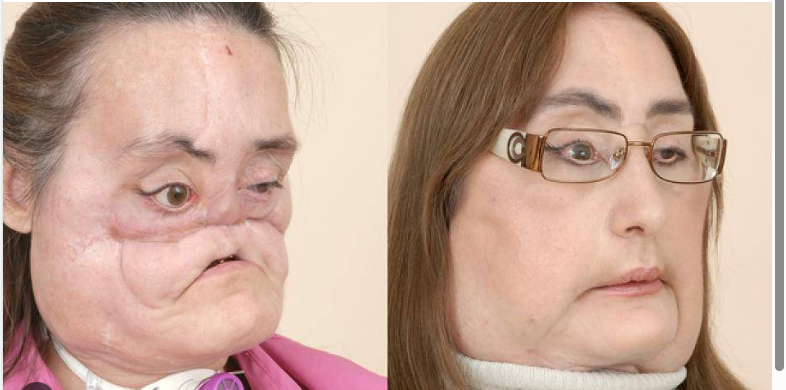


- 1. 1
  2. 2
  3. 3
  4. 4
  5. 5
  6. 6
  7. 7
  8. 8
  9. 9
  10. 10
