# Supplementary material for: Public perception of facial vascularized composite allotransplants-insights from a cross-sectional survey of healthy individuals in the USA
Source: JPRAS Open. 2025 Nov 16;48:123–31. doi: 10.1016/j.jpra.2025.11.015 (PMC12719695; doi:10.1016/j.jpra.2025.11.015)
Supplement: Supplementary file 2 [file mmc2.docx]

**Supplementary Digital Content 1.** Answers to all survey questions reported as absolute and relative frequencies.

| 1. What is your Prolific ID | |  |
| --- | --- | --- |
|  |  |  |
| 2. What is your age? (select one) | |  |
|  | 35-44 | 30 (30.0%) |
|  | 25-34 | 23 (23.0%) |
|  | 45-54 | 23 (23.0%) |
|  | 55-64 | 12 (12.0%) |
|  | 18-24 | 7 (7.0%) |
|  | 65+ | 5 (5.0%) |
|  |  |  |
| 3. Select your sex assigned at birth. (select one) | |  |
|  | Female | 67 (67.0%) |
|  | Male | 33 (33.0%) |
|  |  |  |
| 4. What is your gender? (select one) | |  |
|  | Female | 64 (64.0%) |
|  | Male | 33 (33.0%) |
|  | Non-binary/other | 3 (3.0%) |
|  |  |  |
| 5. What is your race/ethnicity? (select one) | |  |
|  | White | 65 (65.0%) |
|  | Black or African-American | 19 (19.0%) |
|  | Asian | 7 (7.0%) |
|  | Hispanic or Latino | 6 (6.0%) |
|  | Other | 3 (3.0%) |
|  |  |  |
| 6. What is your geographic location? (select one) | |  |
|  | South (South Atlantic: Delaware, Maryland, Virginia, West Virginia, North Carolina, South Carolina, Georgia, Florida or East South Central: Kentucky, Tennessee, Alabama, Mississippi or West South Central: Arkansas, Louisiana, Oklahoma, Texas) | 37 (37.0%) |
|  | Midwest (East North Central: Ohio, Indiana, Illinois, Michigan, Wisconsin or West North Central: Minnesota, Iowa, Missouri, North Dakota, South Dakota, Nebraska, Kansas) | 25 (25.0%) |
|  | West (Mountain: Montana, Idaho, Wyoming, Nevada, Utah, Colorado, Arizona, New Mexico or Pacific: Washington, Oregon, California, Alaska, Hawaii) | 20 (20.0%) |
|  | Northeast (Maine, New Hampshire, Vermont, Massachusetts, Rhode Island, Connecticut, New York, Pennsylvania, New Jersey) | 18 (18.0%) |
|  |  |  |
| 7. Have you ever heard of facial Vascularized Composite Allotransplants (FVCAs)? | | |
|  | No | 58 (58.0%) |
|  | Yes, on social media | 21 (21.0%) |
|  | Yes, on television | 12 (12.0%) |
|  | Yes, other | 8 (8.0%) |
|  | Yes, from peer-groups | 1 (1.0%) |
|  |  |  |
| 8. Have you read or seen information about facial transplant procedures? | |  |
|  | No | 53 (53.0%) |
|  | Yes, on social media | 27 (27.0%) |
|  | Yes, on television | 13 (13.0%) |
|  | Yes, other | 6 (6.0%) |
|  | Yes, from peer-groups | 1 (1.0%) |
|  |  |  |
| 9. Have you or someone close to you experienced severe facial trauma or undergone reconstructive surgery? | | |
|  | No | 86 (86.0%) |
|  | Yes | 14 (14.0%) |
|  |  |  |
| 10. Do you have a background in healthcare? | |  |
|  | No | 84 (84.0%) |
|  | Yes | 16 (16.0%) |
|  |  |  |
| 11. Select your educational background. | |  |
|  | Bachelor‘s degree | 47 (47.0%) |
|  | High school or less | 29 (29.0%) |
|  | Graduate or professional degree | 20 (20.0%) |
|  | Medical or healthcare professional | 4 (4.0%) |
|  |  |  |
| 12. Do you have expertise in any of the following fields? (Check all that apply) | | |
|  | General public (no medical background) | 96 (96.0%) |
|  | Plastic and Reconstructive Surgery | 1 (1.0%) |
|  | Plastic and Reconstructive Surgery, Biomedical Engineering | 1 (1.0%) |
|  | Plastic and Reconstructive Surgery, Dermatology, Aesthetic Medicine | 1 (1.0%) |
|  | Plastic and Reconstructive Surgery, Aesthetic Medicine | 1 (1.0%) |
|  |  |  |
| 13. If you required a face transplant to restore function and appearance, what is the maximum amount you would be willing to pay out-of-pocket for the procedure, assuming costs are not covered by insurance? | | |
|  | $1 - $10,000 | 37 (37.0%) |
|  | $10,001 - $50,000 | 30 (30.0%) |
|  | $0 (I would only proceed if fully covered by insurance) | 14 (14.0%) |
|  | $50,001 - $100,000 | 11 (11.0%) |
|  | > $100,000 | 8 (8.0%) |
|  |  |  |
| 14. If you were a candidate for a face transplant, how long would you be willing to wait for a suitable donor and the procedure? | | |
|  | As long as it takes to find the best match | 48 (48.0%) |
|  | 6 months - 1 year | 17 (17.0%) |
|  | 1-3 months | 16 (16.0%) |
|  | 3-6 months | 10 (10.0%) |
|  | 1-2 years | 5 (5.0%) |
|  | <1 month | 4 (4.0%) |
|  |  |  |
| 15. If you were to receive a facial transplant, which aesthetic complications would bother you the most? | | |
|  | An unnatural or artificial appearance | 37 (37.0%) |
|  | Loss of facial expression or muscle movement | 17 (17.0%) |
|  | Swelling or prolonged facial distortion | 14 (14.0%) |
|  | Visible or excessive scarring | 9 (9.0%) |
|  | Facial asymmetry | 8 (8.0%) |
|  | Mismatch in skin tone or texture | 6 (6.0%) |
|  | None of these would bother me | 5 (5.0%) |
|  | Differences in hair growth (e.g. color, texture or patchy areas) | 2 (2.0%) |
|  | Changes in lip or eyelid positioning | 2 (2.0%) |
|  |  |  |
| 16. Would you prefer a face transplant from a donor who closely matches your age, gender, and ethnicity? | | |
|  | Yes, all three factors should match | 72 (72.0%) |
|  | Age and gender should match, but ethnicity is not important | 12 (12.0%) |
|  | Matching is not important to me | 11 (11.0%) |
|  | Only gender should match | 5 (5.0%) |
|  |  |  |
| 17. Which functional complications after a face transplant would concern you the most? (Select up to three) | | |
|  | Difficulty eating or drinking normally, Difficulty breathing, Chronic pain or discomfort | 23 (23.0%) |
|  | Chronic pain or discomfort | 9 (9.0%) |
|  | Loss of ability to smile or express emotions, Difficulty breathing, Chronic pain or discomfort | 5 (5.0%) |
|  | Difficulty speaking clearly, Difficulty eating or drinking normally, Difficulty breathing | 5 (5.0%) |
|  | Loss of ability to smile or express emotions | 4 (4.0%) |
|  | Difficulty speaking clearly, Difficulty eating or drinking normally, Loss of sensation in the face, Loss of ability to smile or express emotions, Difficulty breathing, Chronic pain or discomfort | 4 (4.0%) |
|  | Difficulty breathing | 4 (4.0%) |
|  | Difficulty speaking clearly, Difficulty breathing, Chronic pain or discomfort | 3 (3.0%) |
|  | Difficulty speaking clearly, Difficulty eating or drinking normally, Chronic pain or discomfort | 3 (3.0%) |
|  | Difficulty speaking clearly, Difficulty eating or drinking normally, Difficulty breathing, Chronic pain or discomfort | 3 (3.0%) |
|  | Difficulty speaking clearly, Loss of ability to smile or express emotions, Chronic pain or discomfort | 3 (3.0%) |
|  | None of these would bother me | 3 (3.0%) |
|  | Difficulty speaking clearly, Difficulty eating or drinking normally, Loss of ability to smile or express emotions, Difficulty breathing | 2 (2.0%) |
|  | Difficulty speaking clearly | 2 (2.0%) |
|  | Loss of sensation in the face, Loss of ability to smile or express emotions, Chronic pain or discomfort | 2 (2.0%) |
|  | Loss of ability to smile or express emotions, Difficulty breathing | 2 (2.0%) |
|  | Loss of sensation in the face, Difficulty breathing, Chronic pain or discomfort | 2 (2.0%) |
|  | Difficulty speaking clearly, Difficulty eating or drinking normally, Loss of ability to smile or express emotions, Difficulty breathing, Chronic pain or discomfort | 2 (2.0%) |
|  | Difficulty eating or drinking normally, Loss of sensation in the face | 1 (1.0%) |
|  | Difficulty speaking clearly, Chronic pain or discomfort | 1 (1.0%) |
|  | Difficulty eating or drinking normally, Loss of sensation in the face, Chronic pain or discomfort | 1 (1.0%) |
|  | Difficulty eating or drinking normally | 1 (1.0%) |
|  | Difficulty speaking clearly, Loss of sensation in the face, Loss of ability to smile or express emotions | 1 (1.0%) |
|  | Difficulty speaking clearly, Loss of ability to smile or express emotions, Difficulty breathing | 1 (1.0%) |
|  | Difficulty eating or drinking normally, Loss of ability to smile or express emotions, Chronic pain or discomfort | 1 (1.0%) |
|  | Difficulty speaking clearly, Loss of sensation in the face, Difficulty breathing | 1 (1.0%) |
|  | Loss of sensation in the face, Loss of ability to smile or express emotions | 1 (1.0%) |
|  | Difficulty eating or drinking normally, Loss of sensation in the face, Loss of ability to smile or express emotions | 1 (1.0%) |
|  | Difficulty speaking clearly, Loss of sensation in the face, Chronic pain or discomfort | 1 (1.0%) |
|  | Difficulty speaking clearly, Loss of sensation in the face, Loss of ability to smile or express emotions, Chronic pain or discomfort | 1 (1.0%) |
|  | Difficulty eating or drinking normally, Chronic pain or discomfort | 1 (1.0%) |
|  | Difficulty eating or drinking normally, Loss of ability to smile or express emotions, Difficulty breathing | 1 (1.0%) |
|  | Loss of sensation in the face, Loss of ability to smile or express emotions, Difficulty breathing, Chronic pain or discomfort | 1 (1.0%) |
|  | Difficulty speaking clearly, Difficulty eating or drinking normally, Loss of sensation in the face, Loss of ability to smile or express emotions | 1 (1.0%) |
|  | Difficulty speaking clearly, Difficulty eating or drinking normally, Loss of sensation in the face | 1 (1.0%) |
|  | Difficulty eating or drinking normally, Loss of ability to smile or express emotions | 1 (1.0%) |
|  | Difficulty speaking clearly, Loss of sensation in the face | 1 (1.0%) |
|  |  |  |
| 18. Are you aware of the risks associated with facial transplants, such as rejection or the need for lifelong immunosuppression? | | |
|  | Slightly aware | 33 (33.0%) |
|  | Not at all aware | 32 (32.0%) |
|  | Aware | 24 (24.0%) |
|  | Extremely aware | 6 (6.0%) |
|  | Neutral | 5 (5.0%) |
|  |  |  |
| 19. Would you be willing to take lifelong immunosuppressive medications (therapy that suppresses the immune system) with potential side effects to maintain a facial transplant? | | |
|  | Yes, but only if side effects are mild | 65 (65.0%) |
|  | Yes, regardless of side effects | 19 (19.0%) |
|  | No, the risks outweigh the benefits | 16 (16.0%) |
|  |  |  |
| 20. If you were to receive a face transplant, how important would donor-recipient resemblance be to you? | | |
|  | Somewhat important | 47 (47.0%) |
|  | Extremely important | 32 (32.0%) |
|  | Neutral | 17 (17.0%) |
|  | Not important at all | 4 (4.0%) |
|  |  |  |
| 21. How would you feel about the possibility of facial rejection, which could require additional surgeries or removal of the transplant? | | |
|  | I would be hesitant but might still consider the procedure | 65 (65.0%) |
|  | I would accept the risk and still undergo the procedure | 21 (21.0%) |
|  | I would not undergo the procedure if rejection was a significant possibility | 14 (14.0%) |
|  |  |  |
| 22. If given the option, would you prefer a partial or full facial transplant? | |  |
|  | Whichever option maximizes my quality of life | 57 (57.0%) |
|  | Partial face transplant to minimize surgical risk | 25 (25.0%) |
|  | Full face transplant for optimal function and appearance | 18 (18.0%) |
|  |  |  |
| 23. How important is maintaining your pre-transplant identity when receiving a new face? | | |
|  | Extremely important—I want to look as much like my old self as possible | 41 (41.0%) |
|  | Somewhat important—I’d like some resemblance but accept changes | 35 (35.0%) |
|  | Neutral—I just want a functional and natural-looking face | 19 (19.0%) |
|  | Not important—I would be fine with a completely different appearance | 5 (5.0%) |
|  |  |  |
| 24. Would you feel comfortable sharing your face transplant journey publicly (e.g., media interviews, social media, medical studies)? | | |
|  | Yes, I would be open to sharing my story | 39 (39.0%) |
|  | Only in medical or scientific settings | 36 (36.0%) |
|  | No, I would prefer to keep it private | 25 (25.0%) |
|  |  |  |
| 25. If you underwent a face transplant, how concerned would you be about societal acceptance and public reactions? | | |
|  | Somewhat concerned—I anticipate challenges but would adjust | 44 (44.0%) |
|  | Extremely concerned—I worry about stigma and negative reactions | 27 (27.0%) |
|  | Neutral—I don’t think it would be a major issue | 18 (18.0%) |
|  | Not concerned at all—I would embrace my new appearance | 11 (11.0%) |
|  |  |  |
| 26. If you needed a face transplant, how much influence would your family or loved ones have on your decision? | | |
|  | Somewhat—I would consider their opinions but make my own choice | 43 (43.0%) |
|  | A great deal—they would need to support the decision | 33 (33.0%) |
|  | Very little—I would decide independently | 17 (17.0%) |
|  | None—my decision would be entirely my own | 7 (7.0%) |
|  |  |  |
| 27. If you required a face transplant, how much of a priority would restoring facial sensation be compared to appearance? | | |
|  | Both are equally important | 47 (47.0%) |
|  | Appearance is more important than sensation | 32 (32.0%) |
|  | Sensation is more important than appearance | 21 (21.0%) |
|  |  |  |
| 28. How does symmetry of facial movement impact the perception of success in a facial transplant? | | |
|  | Some asymmetry is acceptable if function is restored | 51 (51.0%) |
|  | Critical—both static and dynamic symmetry must be achieved | 45 (45.0%) |
|  | Movement symmetry is secondary to overall facial volume and contour | 4 (4.0%) |
|  |  |  |
| 29. How concerned would you be about developing psychological or identity challenges after a face transplant? | | |
|  | Somewhat concerned—I anticipate an adjustment period | 51 (51.0%) |
|  | Extremely concerned—it would be difficult to adjust | 31 (31.0%) |
|  | Neutral—I would take it as it comes | 13 (13.0%) |
|  | Not concerned at all—I would fully embrace the change | 5 (5.0%) |
|  |  |  |
| 30. Would you be willing to undergo multiple surgeries over several years to optimize the outcome of your face transplant? | | |
|  | Yes, but I would prefer to limit the number of procedures | 52 (52.0%) |
|  | Yes, as many as necessary to achieve the best result | 33 (33.0%) |
|  | No, I would want to minimize additional surgeries | 15 (15.0%) |
|  |  |  |
| 31. If a face transplant was your only option to restore function and appearance, but it was considered experimental with unknown long-term outcomes, would you proceed? | | |
|  | Maybe, depending on the available research and expert recommendations | 66 (66.0%) |
|  | Yes, I would take the risk | 21 (21.0%) |
|  | No, I would not want to undergo an experimental procedure | 13 (13.0%) |
|  |  |  |
| 32. What aspect of a face transplant would be the most important in your decision-making? | | |
|  | Restoring normal function (e.g., eating, speaking, breathing) | 51 (51.0%) |
|  | Achieving a natural appearance | 24 (24.0%) |
|  | Minimizing risk of rejection and complications | 14 (14.0%) |
|  | Avoiding lifelong immunosuppressive medications | 7 (7.0%) |
|  | The psychological and social impact | 4 (4.0%) |
|  |  |  |
| 33. Would you consider a face transplant if it meant you would never fully regain facial movement? | | |
|  | Yes, but I would hope for some movement recovery | 60 (60.0%) |
|  | No, I would not undergo the procedure without functional improvement | 30 (30.0%) |
|  | Yes, appearance alone would be enough | 10 (10.0%) |
|  |  |  |
| 34. If a 3D-printed or bioengineered alternative to a face transplant became available, would you prefer it over a donor transplant? | | |
|  | Yes, if it reduces risks like rejection and immunosuppression | 49 (49.0%) |
|  | I would consider whichever option had the best long-term outcomes | 42 (42.0%) |
|  | No, I would prefer a donor face for a more natural result | 9 (9.0%) |
|  |  |  |
| 35. How much does the potential for long-term complications (e.g., chronic rejection, infections) influence your willingness to undergo a face transplant? | | |
|  | Somewhat—I would proceed with caution | 51 (51.0%) |
|  | Significantly—I would be hesitant due to the risks | 38 (38.0%) |
|  | Not much—I would accept the risks for the benefits | 8 (8.0%) |
|  | Not at all—The benefits outweigh the potential risks | 3 (3.0%) |
|  |  |  |
| 36. Would you be willing to relocate to receive a face transplant at a specialized center if it was not available in your current location? | | |
|  | Yes, I would relocate anywhere necessary | 43 (43.0%) |
|  | Yes, but only within a reasonable distance | 42 (42.0%) |
|  | No, I would only undergo the procedure if it was available locally | 15 (15.0%) |
|  |  |  |
| 37. How much of a role should ethical considerations (e.g., donor consent, fairness in organ allocation) play in deciding whether someone receives a face transplant? | | |
|  | A major role—ethical concerns should heavily influence decisions | 61 (61.0%) |
|  | Some role—ethics are important but not the main deciding factor | 27 (27.0%) |
|  | A minor role—the medical need should take priority | 7 (7.0%) |
|  | No role—I would not consider ethical concerns in my decision | 5 (5.0%) |
|  |  |  |
| 38. How important is it for face transplant recipients to receive psychological counseling before and after surgery? | | |
|  | Extremely important—it should be required for all recipients | 82 (82.0%) |
|  | Somewhat important—it should be offered but not required | 16 (16.0%) |
|  | Not important—I don’t think it’s necessary | 2 (2.0%) |
|  |  |  |
| 39. Would you be willing to have your transplanted face appear noticeably different from your original appearance if it meant better function? | | |
|  | Maybe, but I would prefer some resemblance to my original face | 56 (56.0%) |
|  | Yes, function is more important than appearance | 33 (33.0%) |
|  | No, I would not want a face that looks too different | 11 (11.0%) |
|  |  |  |
| 40. If face transplants became more common and widely accepted, how do you think public perception of recipients would change? | | |
|  | They would still face stigma and curiosity | 54 (54.0%) |
|  | They would become more accepted and normalized | 44 (44.0%) |
|  | There would be no significant change in public perception | 2 (2.0%) |
|  |  |  |
| 41. How much influence should surgeons have in deciding whether someone qualifies for a face transplant? | | |
|  | There should be shared decision-making between surgeons and patients | 69 (69.0%) |
|  | Surgeons should have the final say based on medical criteria | 26 (26.0%) |
|  | Patients should have the final say, even if they don’t meet all medical criteria | 5 (5.0%) |
|  |  |  |
| 42. If you underwent a face transplant, how would you prefer to see yourself for the first time? | | |
|  | With medical professionals to guide the experience | 40 (40.0%) |
|  | Alone, in private | 34 (34.0%) |
|  | With close family or friends for support | 26 (26.0%) |
|  |  |  |
| 43. Which of the following factors do you think would matter the most for a successful facial transplant outcome? | | |
|  | Symmetry and alignment | 48 (48.0%) |
|  | Skin tone matching | 21 (21.0%) |
|  | Facial volume (fullness and shape) | 16 (16.0%) |
|  | Scar visibility | 10 (10.0%) |
|  | Skin texture | 5 (5.0%) |
|  |  |  |
| 44. How important is it to you that the skin tone of the transplanted face closely matches the recipient’s original skin tone? (Scale of 1-5, where 1 = Not important, 5 = Extremely important) | | |
|  | 5 | 39 (39.0%) |
|  | 4 | 36 (36.0%) |
|  | 3 | 18 (18.0%) |
|  | 2 | 4 (4.0%) |
|  | 1 | 3 (3.0%) |
|  |  |  |
| 45. How important is achieving natural facial volume (e.g., avoiding puffiness or hollowness) in facial transplants? (Scale of 1-5, where 1 = Not important, 5 = Extremely important) | | |
|  | 4 | 33 (33.0%) |
|  | 5 | 32 (32.0%) |
|  | 3 | 26 (26.0%) |
|  | 2 | 7 (7.0%) |
|  | 1 | 2 (2.0%) |
|  |  |  |
| 46. Do you think slight asymmetry (e.g., one side of the face is slightly different from the other) affects the perceived success of a facial transplant? | | |
|  | Agree | 42 (42.0%) |
|  | Neutral | 27 (27.0%) |
|  | Strongly agree | 20 (20.0%) |
|  | Oppose | 9 (9.0%) |
|  | Strongly Oppose | 2 (2.0%) |
|  |  |  |
| 47. Do you think it’s more important for a facial transplant to focus on: | |  |
|  | Both equally | 66 (66.0%) |
|  | Functionality (ability to smile, speak, eat) | 25 (25.0%) |
|  | Appearance (aesthetics) | 9 (9.0%) |
|  |  |  |
| 48. Would you consider a face transplant as a valid option for individuals with severe facial deformities or injuries? | | |
|  | Yes, it provides a better quality of life | 49 (49.0%) |
|  | Maybe, depending on the results | 49 (49.0%) |
|  | No, I think it’s too risky | 2 (2.0%) |
|  |  |  |
| 49. What are your thoughts on the use of facial transplants to help restore the appearance of individuals with severe trauma, burns, or congenital deformities? | | |
|  | Strongly support | 57 (57.0%) |
|  | Support | 36 (36.0%) |
|  | Neutral | 7 (7.0%) |
|  |  |  |
| 50. How do you feel about the use of facial transplants for purely cosmetic reasons rather than medical necessity? | | |
|  | Neutral | 30 (30.0%) |
|  | Oppose | 26 (26.0%) |
|  | Strongly oppose | 23 (23.0%) |
|  | Support | 17 (17.0%) |
|  | Strongly support | 4 (4.0%) |
|  |  |  |
| 51. If you needed a facial transplant, how important would it be to you that the donor face closely matches your original appearance? (Scale of 1-5, where 1 = Not important, 5 = Extremely important) | | |
|  | 5 | 36 (36.0%) |
|  | 4 | 28 (28.0%) |
|  | 3 | 25 (25.0%) |
|  | 2 | 7 (7.0%) |
|  | 1 | 4 (4.0%) |
|  |  |  |
| 52. How comfortable would you feel interacting with someone who has undergone a facial transplant? (Scale of 1-5, where 1 = Not comfortable and 5 = Very comfortable) | | |
|  | 5 | 46 (46.0%) |
|  | 4 | 31 (31.0%) |
|  | 3 | 16 (16.0%) |
|  | 2 | 4 (4.0%) |
|  | 1 | 3 (3.0%) |
|  |  |  |
| 53. In your opinion, how well do facial transplant patients reintegrate into society after their surgery? | | |
|  | Somewhat well | 44 (44.0%) |
|  | Unsure | 33 (33.0%) |
|  | Not well | 14 (14.0%) |
|  | Very well | 9 (9.0%) |
|  |  |  |
| 54. How important do you think public education is in helping the general population understand facial transplants? | | |
|  | Very important | 45 (45.0%) |
|  | Important | 34 (34.0%) |
|  | Neutral | 16 (16.0%) |
|  | Not important | 5 (5.0%) |
|  |  |  |
| 55. Which aspect of a facial transplant do you think would be the biggest challenge for the recipient? | | |
|  | Psychological adjustment to a new face | 32 (32.0%) |
|  | Risk of rejection and medical complications | 28 (28.0%) |
|  | Social acceptance | 23 (23.0%) |
|  | Functional aspects (e.g., speech, eating, blinking) | 17 (17.0%) |
|  |  |  |
| 56. Which of the following post-transplant facial movement deficits would be most concerning? | | |
|  | Inability to fully close eyes (lagophthalmos) | 48 (48.0%) |
|  | Difficulty with speech articulation | 32 (32.0%) |
|  | Limited smile and perioral movement | 16 (16.0%) |
|  | Lack of forehead or eyebrow mobility | 4 (4.0%) |
|  |  |  |
| 57. Would you perceive a transplanted face as more ‘normal’ if it maintains the ability to express subtle emotions? | | |
|  | Agree | 57 (57.0%) |
|  | Strongly agree | 21 (21.0%) |
|  | Neutral | 20 (20.0%) |
|  | Oppose | 2 (2.0%) |
|  |  |  |
| 58. What level of skin texture difference between transplanted and native facial skin is acceptable? | | |
|  | Mild roughness or scarring is acceptable | 46 (46.0%) |
|  | Moderate differences are acceptable if pigmentation is uniform | 34 (34.0%) |
|  | No noticeable difference | 16 (16.0%) |
|  | Texture mismatches are not a major concern | 4 (4.0%) |
|  |  |  |
| 59. What do you think is the greatest ethical challenge in facial transplantation? | | |
|  | Risk of immunosuppression and long-term complications | 35 (35.0%) |
|  | Identity and psychological adjustment for the recipient | 32 (32.0%) |
|  | Donor selection and consent process | 26 (26.0%) |
|  | Social stigma and public perception | 7 (7.0%) |
|  |  |  |
| 60. How successful do you rate the results of the operation? (1 completely inadequate, 10 perfect)  Link: https://de.pinterest.com/pin/510384570247778055/  (Source: CBS News) | | |
|  | 8 | 32 (32.0%) |
|  | 9 | 29 (29.0%) |
|  | 10 | 23 (23.0%) |
|  | 7 | 10 (10.0%) |
|  | 6 | 3 (3.0%) |
|  | 5 | 3 (3.0%) |
|  |  |  |
| 61. How successful do you rate the results of the operation? (1 completely inadequate, 10 perfect)  Link: https://hospitalnews.com/first-canadian-face-transplant-a-success/  (Source: Hospital News) | | |
|  | 8 | 32 (32.0%) |
|  | 9 | 31 (31.0%) |
|  | 7 | 17 (17.0%) |
|  | 10 | 17 (17.0%) |
|  | 5 | 1 (1.0%) |
|  | 4 | 1 (1.0%) |
|  | 6 | 1 (1.0%) |
|  |  |  |
| 62. How successful do you rate the results of the operation? (1 completely inadequate, 10 perfect)  Link: https://www.3d-grenzenlos.de/magazin/kurznachrichten/3d-druck-macht-gesichtstransplantation-moeglich-27233393/  (Source: 3D GRENZENLOS) | | |
|  | 8 | 35 (35.0%) |
|  | 9 | 22 (22.0%) |
|  | 7 | 19 (19.0%) |
|  | 10 | 13 (13.0%) |
|  | 6 | 8 (8.0%) |
|  | 5 | 3 (3.0%) |
|  |  |  |
| 63. How successful do you rate the results of the operation? (1 completely inadequate, 10 perfect)  Link: https://www.plasticsurgery.org/for-medical-professionals/publications/psn-extra/news/planning-the-key-to-highly-complicated-and-successful-face-transplant  (Source: American Society of Plastic Surgeons) | | |
|  | 10 | 26 (26.0%) |
|  | 9 | 25 (25.0%) |
|  | 8 | 22 (22.0%) |
|  | 7 | 20 (20.0%) |
|  | 5 | 4 (4.0%) |
|  | 6 | 2 (2.0%) |
|  | 2 | 1 (1.0%) |
|  |  |  |
| 64. How successful do you rate the results of the operation? (1 completely inadequate, 10 perfect)  Image 5 Link: https://www.faz.net/aktuell/gesellschaft/gesundheit/gesichtstransplantation-ich-habe-meine-nase-wieder-1795266.html  (Source: Frankfurter Allgemeine) | | |
|  | 6 | 27 (27.0%) |
|  | 7 | 20 (20.0%) |
|  | 8 | 18 (18.0%) |
|  | 5 | 14 (14.0%) |
|  | 9 | 6 (6.0%) |
|  | 4 | 6 (6.0%) |
|  | 10 | 5 (5.0%) |
|  | 3 | 2 (2.0%) |
|  | 2 | 2 (2.0%) |
